# Physical Activity Types, Physical Activity Levels and Risk of Diabetes in General Adults: The NHANES 2007–2018

**DOI:** 10.3390/ijerph20021398

**Published:** 2023-01-12

**Authors:** Chunnan Li, Shaomei Shang, Wannian Liang

**Affiliations:** 1 Vanke School of Public Health, Tsinghua University, Beijing 100084, China; 2 Institute for Healthy China, Tsinghua University, Beijing 100084, China; 3 School of Nursing, Peking University, Beijing 100191, China

**Keywords:** diabetes, physical activity, epidemiology, NHANES

## Abstract

Background: Recreational activities show benefits for diabetes prevention, but work-related activity and the total amount of individual physical activity is rarely discussed. Purpose: The purpose of this study was to evaluate the participation in five typical physical activities (vigorous work activity, vigorous recreational activities, moderate work activity, moderate recreational activities, and walk/bicycle for transportation), as well as the weekly distribution of total physical activity intensity, and to explore the relationships between physical activity types, physical activity levels, and risk of diabetes. Study design: Cross-sectional study. Methods: The self-reported physical activity data on specific domains of physical activity were acquired from individuals in the 2007–2018 National Health and Nutrition Examination Survey (NHANES) using the Physical Activity Questionnaire (PAQ). Diabetes status was assessed by self-reported medical diagnosis or medication usage, or a fasting glucose concentration ≥ 126 mg/dL (fasting is defined as no caloric intake for at least 8 h) or HbA1c ≥ 6.5%. Weighted logistic regression was used to investigate the associations between physical activity types, physical activity levels, and risk of diabetes. Results: Diabetes was less prevalent in people who participated in physical activity and the risk of diabetes reduced progressively as total physical activity levels increased. Younger adults (20–44 years) and males reported a higher proportion of high-intensity physical activity participation. Conclusions: Our findings highlight the importance of a physically active lifestyle for preventing diabetes. Distinct types of physical activity had different effects on the risk of diabetes. A greater total physical activity level was related to a substantial reduction in diabetes risk.

## 1. Introduction

Diabetes affects almost half a billion people worldwide, accounting for more than 10.5% of the adult population and an increasing trend is anticipated to continue, posing a serious danger to global health [1]. According to the recent National Health and Nutrition Examination Survey (NHANES) analysis, the unadjusted prevalence of total diabetes increased from 7.7% in 1999–2000 to 13.3% in 2015–2016 in general U.S. adults [2].

Epidemiological studies and diabetes prevention trials have recognized the importance of lifestyle in diabetes risk, and physical activity is one of the effective lifestyle behaviors [3,4,5]. The majority of prior research consistently found that physical activity was associated with a reduced risk of diabetes [6,7,8,9,10], but the relationship differed somewhat among studies. A systematic review and meta-analysis revealed a decrease in risk of diabetes incidence with rising levels of physical activity [11]. The Whitehall II cohort discovered that moderate-to-vigorous physical activity had a significant role in diabetes, impacting both incidence and prognosis [12]. A study based on the Survey of Health, Ageing, and Retirement in Europe found that moderate physical activity can reduce the risk of diabetes [13]. In contrast, another study based on Japanese employees found that, in addition to moderate physical activity, only vigorous physical activity can lower the incidence of diabetes [14]. Furthermore, two meta-analyses revealed that recreational physical activity, as well as occupational physical activity, were inversely related to diabetes risk [15,16]. Conversely, some studies suggested that there was no association between leisure time physical activity and diabetes risk among people with high occupational physical activity or low occupational physical activity with movement [17], as well as among those who walked to and from work [14].

Thus, although many studies have shown that an active lifestyle can lower the risk of diabetes [18,19], most research has focused exclusively on leisure-time physical activities. There was limited evidence that different types of physical activities had the same health-improving effects and no solid evidence to support recommendations regarding the required physical activity intensity for each type of physical activity. Therefore, based on the nationally representative survey, we aimed to examine the association between each physical activity type (vigorous work activity, vigorous recreational activities, moderate work activity, moderate recreational activities, and walk/bicycle) and physical activity intensity (low, moderate, and high) with the risk of diabetes.

## 2. Materials and Methods

### 2.1. Study Population

The NHANES is a nationally representative cross-sectional survey undertaken by the Centers for Disease Control and Prevention that is based on a stratified multistage random sample methodology. The questionnaire data were collected at the respondents’ homes by professional interviewers. Blood was collected in the mobile examination center (MEC) by a phlebotomist, and the refrigerated or frozen samples were transported to laboratories for testing. The physical examinations were also conducted at the MEC. Data from the NHANES 2007–2008, 2009–2010, 2011–2012, 2013–2014, 2015–2016 and 2017–2018 cycles were used in this analysis.

Among the 59,842 participants from NHANES 2007–2018, participants under the age of 20 were the first to be excluded (*n* = 25,072), followed by those who were pregnant or unable to ascertain (females in 20–44 years, *n* = 720). Participants who were unable to determine if they had diabetes were excluded (*n* = 210) according to the definition. NHANES examined the physical activity data for completeness, consistency, and logic. In this study, we carefully examined the data and excluded physical activity and sedentary time information that was missing, rejected, or unknown, as well as total time of physical activity, sedentary time, and sleep duration that was greater than 24 h (*n* = 643). Furthermore, participants with missing, rejected, or unknown covariates were excluded (*n* = 1867). Finally, 31,330 participants with no missing or confusing information regarding the main outcome, exposure, or variables were included.

### 2.2. Definition of Diabetes

Participants were considered to have diabetes for any of the following reasons: if they responded “yes” to the question “Have you ever been told by a doctor or health professional that you have diabetes or sugar diabetes?”; if they self-reported taking insulin or diabetic pills; or if they had a fasting glucose concentration ≥126 mg/dL (fasting is defined as no caloric intake for at least 8 h) or HbA1c ≥ 6.5% [20].

### 2.3. Assessment of Physical Activity

The Global Physical Activity Questionnaire was used to collect respondent-level weekly physical activity information [21]. Physical activity levels were examined for three categories of physical activity participation: vigorous work activity/vigorous recreational activities, moderate work activity/moderate recreational activities, and walk/bicycle information. Vigorous work activity was defined as typical activity that induced significant increases in breathing or heart rate for at least 10 min continuously, such as hauling or lifting heavy weights, excavating, or building work. Vigorous recreational activities were characterized as high-intensity sports, fitness activities, or leisure activities that generate significant increases in respiration or heart rate, such as jogging or basketball. Moderate work activity was defined as any activity that induces minor increases in breathing or heart rate, such as brisk walking or carrying light burdens for at least 10 min continuously. Moderate leisure activities were defined as those that generate a slight rise in breathing or heart rate for at least 10 min continuously, such as brisk walking, biking, swimming, or volleyball. Walking or use of a bicycle was defined as a transportation to school/work or for shopping for at least 10 min continuously to get to and from places.

The weekly metabolic equivalent (MET) was determined using the NHANES suggested MET scores of 8 points for vigorous work activity/vigorous recreational activities and 4 points for moderate work activity/moderate recreational activities and walk/bicycle. The weekly metabolic equivalent of the physical activity was computed using the MET scores of the physical activity engaged in, the daily duration, and the number of days per week they were engaged in, and then physical intensity was classified into four categories based on their quartiles of total metabolic equivalent. The tertiles of physical activity levels were pooled in the subgroup analysis of physical activity intensity (>0 met-h/week) for each physical activity type (From Quartile 1 to Quartile 3/Quartile 4).

### 2.4. Assessment of Covariates

The following information was gathered using a household structured questionnaire: gender (male, female), age (20–44, 45–64, ≥65 years), education level (less than 9th grade, 9–11th grade, 12th grade with no diploma, high school graduate/GED or equivalent, some college or AA degree, college graduate, or above), marital status (married/living with partner, widowed, divorced, separated, never married), race (Mexican American, other Hispanic, non-Hispanic Black, other race—including multi-racial, non-Hispanic White), type of work done last week (working at a job or business, with a job or business but not at work, looking for work, not working at a job or business). Sedentary time refers to the amount of time spent sitting in a normal day, excluding sleep. Smoking status was determined by asking the question, “Have you smoked at least 100 cigarettes in your whole life?” (yes/no). BMI was determined as weight (kg) divided by height squared (kg/m^2^). Participants were defined as hypertensive based on self-reported medical diagnosis, antihypertensive medicine usage, or a high blood pressure measurement value (systolic blood pressure 140 mm Hg and/or diastolic blood pressure 90 mm Hg). CVD was characterized as having one or more of the following diseases: congestive heart failure, coronary heart disease, angina pectoris, heart attack, and stroke.

### 2.5. Statistical Analysis

The participants’ baseline characteristics were classified based on their level of physical activity and diabetes status. A descriptive statistical analysis was performed on basic demographic information (gender, age, education level, race, marital status), behavioral aspects (vigorous work activity, vigorous recreational activities, moderate work activity, moderate recreational activities, walk or bicycle, physical activity levels, sedentary time), body measurements (BMI), and diseases (hypertension, CVD). The categorical variables were reported in frequency (%), with the chi-square test for unordered categorical variables and the chi-square trend test for ordered multi-categorical variables.

We used the mobile examination center exam weight in the analyses to account for the cluster sample design, oversampling, poststratification, survey nonresponse, and sampling frame, as advised. The survey’s weight permits it to be expanded to the civilian noninstitutionalized US population [22,23,24]. Weighted logistic regression was performed to obtain odds ratios (ORs) and 95% confidence intervals (CIs) to explore the association between physical activity types/levels and risk of diabetes. The same procedure also applied to subgroup analysis. Q1 is the control group in the overall, gender, and age subgroup analyses, while no activity (0 met-h/week) is the control group in the physical activity types subgroup analysis.

STATA version 14.0 was used for statistical analysis (Stata Corp LP, College Station, TX, USA). R version 3.5.3 was used to create the forest graphs. A *p*-value under 0.05 was considered statistically significant.

## 3. Results

Among the 31,330 participants (48.8% males and 51.2% females, mean (SD) age, 50.1 [18.1] years), the prevalence of diabetes was 17.5%. Table 1 displays the baseline characteristics of the participants based on diabetes status. Overall, diabetes was more common in middle-aged (45–64 years) and elderly (≥65 years) people, males, and non-whites. Participants who were less education, possible unemployed, living a sedentary lifestyle, heavy smokers, and in a poor metabolic condition, such as being overweight or suffering from hypertension or CVD, had a higher prevalence of diabetes. Diabetes was less frequent in individuals who engaged in physical activity, especially vigorous recreational activities, and the prevalence of diabetes decreased significantly as physical activity intensity increased (Table 1). Furthermore, participants who engaged in higher intensity physical activity had a lower BMI and were less likely to have hypertension or cardiovascular disease (Table 2). Approximately 26.9% of the study population did not participate in any form of physical activity. Young individuals (20–44 years), males, non-whites, and participants with employment were more likely to be engaged in higher intensity physical activity (Table 2).

**Figure 1:**
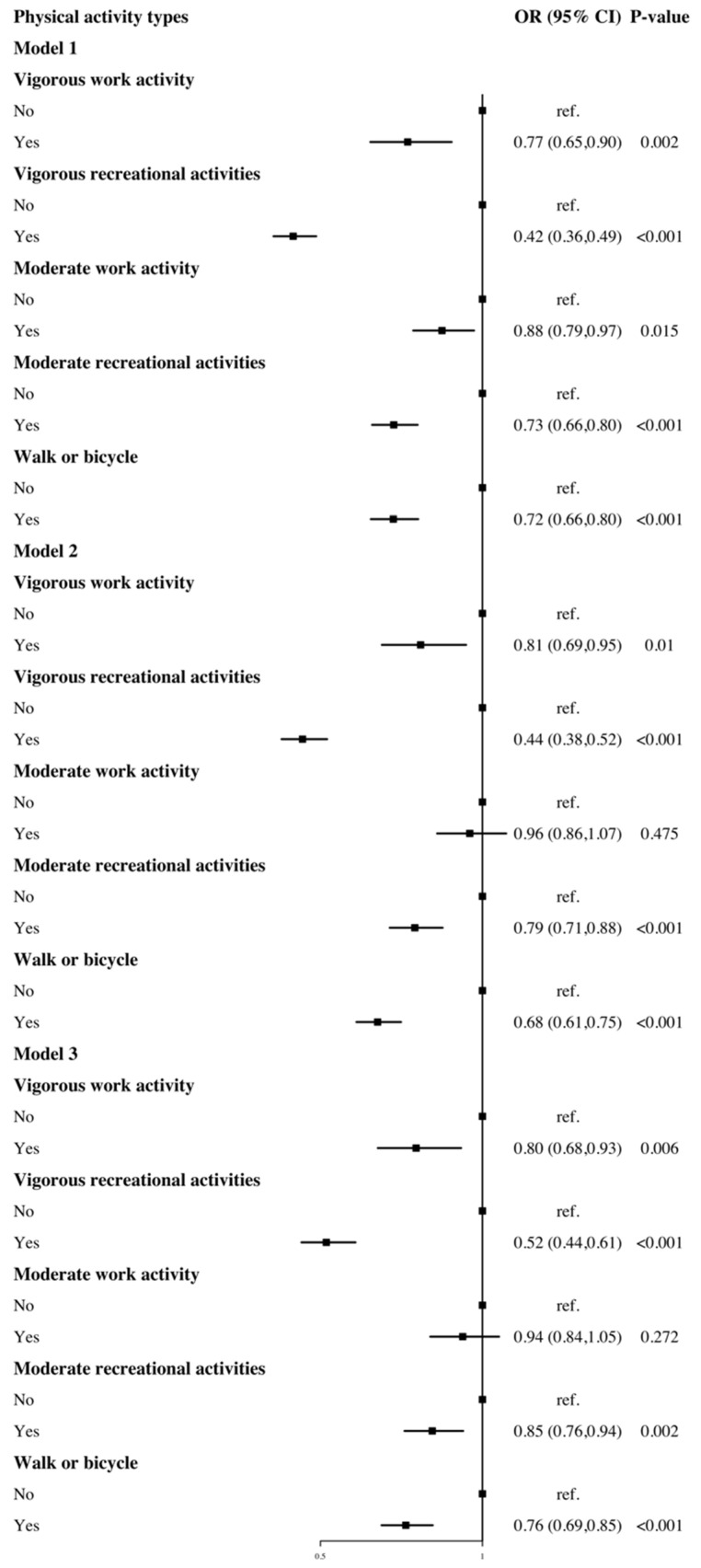
The association between physical activity types and risk of diabetes. Model 1 adjusted for gender, age; Model 2 adjusted for gender, age, race, marital status, education level, occupation, smoking status, sedentary time. Model 3 adjusted for gender, age, race, marital status, education level, occupation, smoking status, sedentary time, BMI, hypertension, CVD.

Figure 1 presents the association between involvement in various types of physical activity and diabetes risk. Overall, all types of physical activity considered in this study were associated with a lower risk of diabetes; non-work-related physical activity, particularly vigorous recreational activities, had the strongest statistical correlation (Model 1, OR: 0.42, 95% CI: 0.36 to 0.49). Except for moderate work activity (Model 3, OR: 0.94, 95% CI: 0.84 to 1.05), all correlations remained statistically significant after controlling for gender, age, race, marital status, education level, occupation, smoking status, sedentary time, BMI, hypertension, and CVD (in the fully adjusted models).

**Figure 2:**
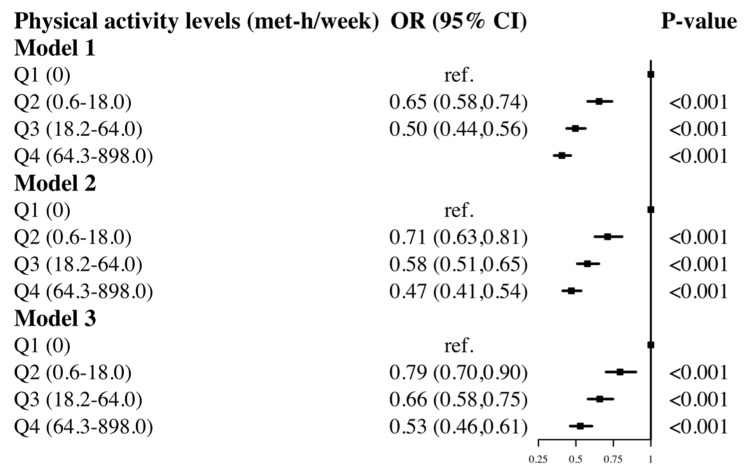
The association between physical activity levels and risk of diabetes. Model 1 adjusted for gender, age; Model 2 adjusted for gender, age, race, marital status, education level, occupation, smoking status, sedentary time. Model 3 adjusted for gender, age, race, marital status, education level, occupation, smoking status, sedentary time, BMI, hypertension, CVD.

Figure 2 shows that the risk of diabetes decreased as the total intensity of physical activity increased across the three models. Diabetes risks were decreased by 21% (OR: 0.79, 95% CI: 0.70 to 0.90), 34% (OR: 0.66, 95% CI: 0.58 to 0.75), and 47% (OR: 0.53, 95% CI: 0.46 to 0.61) in those who engaged in 0.6–18.0 met-h/week, 18.2–64.0 met-h/week, and 64.3–898.0 met-h/week physical activity, respectively, compared to no physical activity (model 3). The inverse dose–response relationship between total physical intensity and diabetes risk varied with age and gender. Low-intensity physical activity (0.6–18.0 met-h/week, OR: 0.77, 95% CI: 0.57 to 1.04) was not significantly related with diabetes risk in young individuals (20–44 years), while moderate (18.2–64.0 met-h/week, OR: 0.53, 95% CI: 0.39 to 0.72) to high (64.3–898.0 met-h/week, OR: 0.54, 95% CI: 0.42 to 0.71) levels of physical activity had similar diabetes preventive benefits and were associated with decreased risk of diabetes (Supplementary Figure S1A). In middle-aged persons (45–64 years), the higher the intensity of physical activity, the lower the risk of diabetes (0.6–18.0 met-h/week, OR: 0.81, 95% CI: 0.65 to 0.99; 18.2–64.0 met-h/week, OR: 0.64, 95% CI: 0.52 to 0.80; 64.3–898.0 met-h/week, OR: 0.51, 95% CI: 0.40 to 0.64, Supplementary Figure S1B). In the elderly (≥65 years), the effects of intermediate and low intensity were similar, whereas high intensity was linked to a decreased incidence of diabetes (0.6–18.0 met-h/week, OR: 0.77, 95% CI: 0.64 to 0.92; 18.2–64.0 met-h/week, OR: 0.77, 95% CI: 0.63 to 0.94; 64.3–898.0 met-h/week, OR: 0.54, 95% CI: 0.43 to 0.69, Supplementary Figure S1C). In the gender stratification, both men and women showed that the risk of diabetes decreased with increasing physical activity intensity, with the preventative effect for men somewhat greater than for women (Supplementary Figure S2A,B).

**Figure 3:**
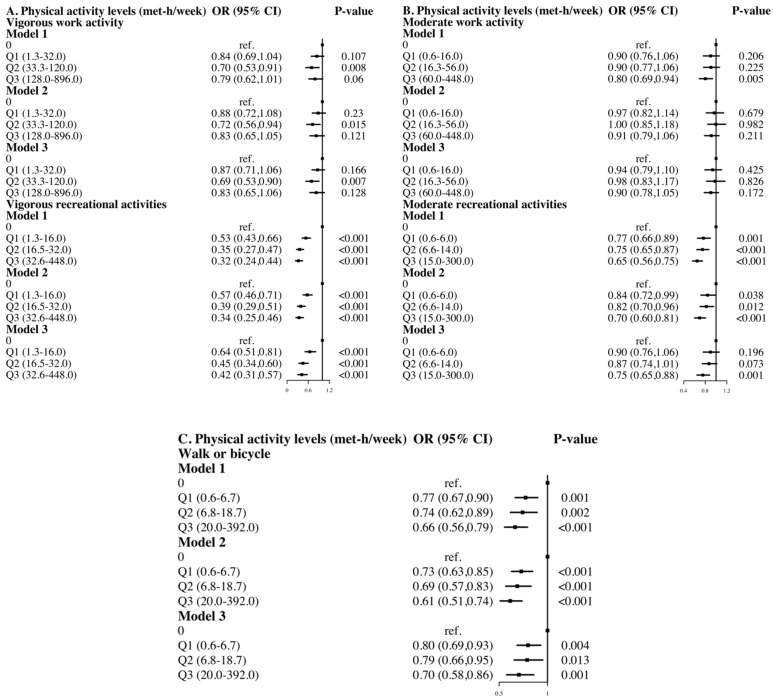
The association between levels of each physical activity and risk of diabetes. Model 1 adjusted for gender, age; Model 2 adjusted for gender, age, race, marital status, education level, occupation, smoking status, sedentary time. Model 3 gender, age, race, marital status, education level, occupation, smoking status, sedentary time, BMI, hypertension, CVD.

As shown in Figure 3A–C, among five different domains of physical activity, including vigorous work activity, vigorous recreational activities, moderate work activity, moderate recreational activities, and walk/bicycle, the three degrees of physical activity levels had differential effects on diabetes risk compared to inactive responders. Participants who engaged in moderate-intensity vigorous work activity (OR: 0.69, 95% CI: 0.53 to 0.90) had a considerably decreased risk of diabetes, while neither higher (OR: 0.83, 95% CI: 0.65 to 1.06) nor lower (OR: 0.87, 95% CI: 0.71 to 1.06) level of physical activity showed no significant link with diabetes risk (Figure 3A). For vigorous recreational activities, on the other hand, the higher the levels of activity, the lower the risk of diabetes (Q1-OR: 0.64, 95% CI: 0.51 to 0.81; Q2-OR: 0.45, 95% CI: 0.34 to 0.60; Q3-OR: 0.42, 95% CI: 0.31 to 0.57, Figure 3A). The level of moderate recreational activities was adversely connected with the risk of diabetes, which was equivalent to the relationship between vigorous recreational activities and diabetes, but a significant difference was detected only in the population with the highest level (Q3-OR: 0.75, 95% CI: 0.65 to 0.88, model 3, Figure 3B). In terms of transportation-related walking or cycling, the higher the levels, the lower the risk of diabetes (Q1-OR: 0.80, 95% CI: 0.69 to 0.93; Q2-OR: 0.79, 95% CI: 0.66 to 0.95; Q3-OR: 0.70, 95% CI: 0.58 to 0.86, Figure 3C).

## 4. Discussion

In this national representative study, all types of physical activity considered were associated with the risk of diabetes, except for moderate work activity, with vigorous recreational activities having the largest protective effect. Overall, increasing weekly metabolic equivalents reduced the risk of diabetes, although not for all types of physical activities, such as work-related physical activity. In addition, the relationship between physical activity levels and diabetes risk varied by sex and age.

Our findings are broadly consistent with prior studies demonstrating that participating in physical activity was related to a decreased prevalence of diabetes. Across the Diabetes Prevention Program study (*n* = 3234), the self-reported and accelerometer-measured physical activity were both inversely related to diabetes risk [5]. Our results were conducted with a larger sample size (*n* = 31,330) of adults (≥20 years), indicating that self-reported physical activity participation was related to a 33% decreased risk of diabetes (OR: 0.67, 95% CI: 0.61 to 0.74), with additional evidence of a physical activity benefit. According to current diabetes preventive guidelines, individuals can lower their risk of diabetes by engaging in a range of physical activities [25]. The PURE study involved 130,000 adults from 17 countries and suggested that both recreational and non-recreational physical activity were associated with cardiovascular health benefits [26]. However, the majority of research has only focused on recreational physical activity benefits, and research on nonrecreational physical activity is scarce [15]. 

Prior evidence has showed that increased free-living physical activity/exercise can considerably improve HbA1c in persons with diabetes [27]. In a Finnish randomized controlled experiment, increased physical activity reduced the incidence of type 2 diabetes in high-risk groups, with a reduced risk of diabetes in response to moderate-to-vigorous leisure-time physical activity of 63–65% [28]. In a prospective cohort of Chinese people with impaired fasting glucose, those who reported low, moderate, and high leisure-time physical activity volume showed a 12%, 20%, and 25% reduction in diabetes risk, respectively, when compared to inactive persons [10]. Additionally, a regular 30-year follow-up study discovered that moderate-to-vigorous intensity physical activity levels in young adulthood and reductions in later adulthood were both substantially and independently linked with diabetes development later in life [29]. Similarly, our findings show that moderate and vigorous recreational physical activities are related to a 15% to 48% decrease in diabetes risk (OR: 0.85, 95% CI: 0.76 to 0.94; OR: 0.52, 95% CI: 0.44 to 0.61, Figure 1). 

Moreover, limited evidence was provided on the association between work-related physical activity and diabetes risk. A prior study using NHANES 2011–2016 data found that, in comparison to not meeting the guideline recommendations, those who achieved adequate physical activity, occupation-, leisure-, and transportation-based physical activity level had a 22%, 16%, 18%, and 22% decreased risk of diabetes, respectively [30]. Otherwise, a U.K. cohort study on self-reported routine physical activity (regular walking and cycling, recreational activity, and sport) discovered that even modest PA significantly reduced diabetes risk [31]. In our study, compared to inactive adults, the results only supported an association between vigorous-intensity work activity and a decreased risk of diabetes (OR: 0.80, 95% CI: 0.68 to 0.93, Figure 1), whereas moderate intensity did not demonstrate a statistically significant association (OR: 0.94, 95% CI: 0.84 to 1.05, Figure 1). Our findings indicate that exercise such as fast walking may not appear to have a substantial diabetes preventive impact, but walking or cycling can lower the diabetes risk by 24% (OR: 0.76, 95% CI: 0.69 to 0.85, Figure 1). A three-year randomized clinical trial in Italy revealed that even small increases in moderate-to-vigorous intensity physical activities might have a clinically significant impact, presumably by boosting total energy expenditure [32]. In our study, increased total energy expenditure was associated with a 21%-47% lower risk of diabetes compared to inactive adults (Q2-OR: 0.79, 95% CI: 0.70 to 0.90; Q3-OR: 0.66, 95% CI: 0.58 to 0.75; Q4-OR: 0.53, 95% CI: 0.46 to 0.61). Moreover, several studies have further investigated the ideal amounts of specific exercise to avoid diabetes risk. A cross-sectional study based on a Chinese rural population showed that lower physical activity was significantly associated with diabetes risk in middle-aged and elderly adults [33]. Another Chinese study found that moderate-to-vigorous exercise ≥2250 METs per week was associated with a lower risk of diabetes in the middle-aged group (45–64 years old) [34]. A recent study found that household physical activity level was inversely associated with the risk of diabetes among urban males in northern China [35]. Similarly, we carried out an exploration of the quantity of physical activities in various forms, and showed that compared to being inactive, and vigorous work activity (33.3–120.0 METs per week), such as hauling or lifting heavy weights, excavating, or building work, was associated with the lowest diabetes risk.

There is compelling evidence that comprehensive lifestyle adjustments centered on increasing physical activity might be more beneficial than pharmaceutical therapies in diabetes prevention [36]. A randomized controlled trial suggested that physical activity favorably affects glycemic parameters, lipid profile, blood pressure, and hs-CRP [37]. In addition, physical activity can improve blood lipids and inflammatory indicators. An exercise-induced mild acute increase of IL-6 in humans appears to have a direct anti-inflammatory impact by suppressing TNF- and increasing IL-1ra (an IL-1 receptor antagonist), restricting IL-1 signaling [38]. Additionally, IL-1 has been demonstrated to be implicated in pancreatic cell destruction, whereas tumor necrosis factor seems to be a critical molecule in peripheral insulin resistance and IL-6 has a direct influence on glucose and lipid metabolism [38]. A prospective follow-up study discovered that antidiabetic benefits were mediated by oxidized low-density lipoprotein and total antioxidant capacity, indicating that moderate physical exercise decreases diabetes risk by lowering oxidative stress [39]. Otherwise, the indirect anti-inflammatory benefits of physical activity might be mediated by changes in body composition [38]. For example, an aerobic, resistance, and combined physical activity investigation in obese individuals revealed alterations in cytokine/adipokine levels, as well as improved insulin resistance [40]. Physical activity might be a natural, powerful anti-inflammatory and metabolic-improvement strategy with fewer negative effects.

The strengths of this analysis included the large sample size, a nationally representative design, multiple types of information on both work and recreational physical activities, analyses of overall and subgroups with diabetes risk. Nevertheless, our research still, with limitations, needs to be strengthened. Since the data utilized in the analysis were cross-sectional, causation could not be confirmed. Moreover, as all types and levels of physical activity was based on self-reporting, the recall bias was inevitable. As certain variables have different time frames, the interrelationship between various outcomes may be misinterpreted. Finally, the time span of the analysis data was from 2007 to 2018 and the effect of sociocultural changes on exercise preferences over time was not taken into account.

## 5. Conclusions

In summary, we demonstrated the cross-sectional health impact of leisure-time and occupational physical activities. Future study is needed to examine different types and volumes of physical activity, rather than just leisure activities, for improved diabetes prevention and support.

## Figures and Tables

**Table 1:** Baseline Characteristics of Participants by Diabetes status: NHANES 2007–2018.

Characteristic	Diabetes		
	No (*n* = 25,848)	Yes (*n* = 5482)	*p*-Value
Age (years)			<0.001
20–44	12,288 (94.9)	663 (5.1)	
45–64	8485 (77.6)	2444 (22.4)	
≥65	5075 (68.1)	2375 (31.9)	
Gender			<0.001
Male	12,451 (81.4)	2844 (18.6)	
Female	13,397 (83.5)	2638 (16.5)	
Race			<0.001
Non-white	14,924 (80.3)	3656 (19.7)	
White	10,924 (85.7)	1826 (14.3)	
Marital status			<0.001
Married/Living with partner	15,310 (82.4)	3274 (17.6)	
Widowed/Divorced/Separated	5299 (76.0)	1671 (24.0)	
Never married	5239 (90.7)	537 (9.3)	
Education level			<0.001
≤High school	11,680 (78.9)	3115 (21.1)	
>High school	14,168 (85.7)	2367 (14.3)	
Occupation			<0.001
With a job or business	15,450 (88.3)	2041 (11.7)	
Looking for work/Not working	10,398 (75.1)	3441 (24.9)	
Vigorous work activity ^a^			<0.001
No	20,680 (81.3)	4761 (18.7)	
Yes	5168 (87.8)	721 (12.2)	
Vigorous recreational activities ^b^			<0.001
No	19,489 (79.5)	5031 (20.5)	
Yes	6359 (93.4)	451 (6.6)	
Moderate work activity ^c^			<0.001
No	16,214 (80.6)	3905 (19.4)	
Yes	9634 (85.9)	1577 (14.1)	
Moderate recreational activities ^d^			<0.001
No	15,054 (79.9)	3791 (20.1)	
Yes	10,794 (86.5)	1691 (13.5)	
Walk or bicycle			<0.001
No	19,063 (81.2)	4401 (18.8)	
Yes	6785 (86.3)	1081 (13.7)	
Physical activity levels (met-h/week)			<0.001
Q1 (0)	6242 (74.1)	2185 (25.9)	
Q2 (0.6–18.0)	6049 (81.2)	1398 (18.8)	
Q3 (18.2–64.0)	6652 (85.9)	1094 (14.1)	
Q4 (64.3–898.0)	6905 (89.6)	805 (10.4)	
Sedentary time (hours)			0.005
<5	10,781 (83.2)	2174 (16.8)	
≥5	15,067 (82.0)	3308 (18.0)	
Smoke status			<0.001
No	14,779 (84.1)	2804 (15.9)	
Yes	11,069 (80.5)	2678 (19.5)	
BMI status (kg/m^2^)			<0.001
<25	8312 (92.0)	725 (8.0)	
≥25	17,536 (78.7)	4757 (21.3)	
Hypertension			<0.001
No	16,366 (91.6)	1510 (8.4)	
Yes	9482 (70.5)	3972 (29.5)	
CVD			<0.001
No	23,891 (85.2)	4147 (14.8)	
Yes	1957 (59.4)	1335 (40.6)	

Abbreviations: n: sample size; BMI, body mass index (calculated as weight in kilograms divided by height in square meters); MET, metabolic equivalent; CVD, cardiovascular disease (suffering congestive heart failure, coronary heart disease, angina pectoris, heart attack, or stroke). ^a^ Vigorous work activity: typical activity that induced significant increases in breathing or heart rate for at least 10 min continuously; ^b^ Vigorous recreational activities: high-intensity sports, fitness activities, or leisure activities that generate significant increases in respiration or heart rate; ^c^ Moderate work activity: any activity that induces minor increases in breathing or heart rate; ^d^ Moderate recreational activities: any activity that generates a slight rise in breathing or heart rate for at least 10 min continuously.

**Table 2:** Baseline Characteristics of Participants by Physical Activity Intensity Status: NHANES, 2007–2018.

Characteristic	Physical Activity Levels (met-h/week)				
	Q1 (*n* = 8427)	Q2 (*n* = 7447)	Q3 (*n* = 7746)	Q4 (*n* = 7710)	*p*-Value
Age (years)					<0.001
20–44	2318 (27.5)	2713 (36.4)	3571 (46.1)	4349 (56.4)	
45–64	3048 (36.2)	2826 (37.9)	2597 (33.5)	2458 (31.9)	
≥65	3061 (36.3)	1908 (25.6)	1578 (20.4)	903 (11.7)	
Gender					<0.001
Male	3328 (39.5)	3111 (41.8)	3907 (50.4)	4949 (64.2)	
Female	5099 (60.5)	4336 (58.2)	3839 (49.6)	2761 (35.8)	
Race					<0.001
Non-white	5250 (62.3)	4493 (60.3)	4352 (56.2)	4485 (58.2)	
White	3177 (37.7)	2954 (39.7)	3394 (43.8)	3225 (41.8)	
Marital status					<0.001
Married/Living with partner	4857 (57.6)	4492 (60.3)	4715 (60.9)	4520 (58.6)	
Widowed/Divorced/Separated	2482 (29.5)	1769 (23.8)	1432 (18.5)	1287 (16.7)	
Never married	1088 (12.9)	1186 (15.9)	1599 (20.6)	1903 (24.7)	
Education levels					<0.001
≤High school	4908 (58.2)	3240 (43.5)	2903 (37.5)	3744 (48.6)	
>High school	3519 (41.8)	4207 (56.5)	4843 (62.5)	3966 (51.4)	
Occupation					<0.001
With a job or business	3473 (41.2)	3881 (52.1)	4603 (59.4)	5534 (71.8)	
Looking for work/Not working	4954 (58.8)	3566 (47.9)	3143 (40.6)	2176 (28.2)	
Smoke status					<0.001
No	4643 (55.1)	4358 (58.5)	4569 (59.0)	4013 (52.0)	
Yes	3784 (44.9)	3089 (41.5)	3177 (41.0)	3697 (48.0)	
Sedentary time (hours)					<0.001
<5	2869 (34.0)	2656 (35.7)	2949 (38.1)	4481 (58.1)	
≥5	5558 (66.0)	4791 (64.3)	4797 (61.9)	3229 (41.9)	
BMI status (kg/m^2^)					<0.001
<25	2072 (24.6)	2158 (29.0)	2487 (32.1)	2320 (30.1)	
≥25	6355 (75.4)	5289 (71.0)	5259 (67.9)	5390 (69.9)	
Diabetes					<0.001
No	3193 (37.9)	3355 (45.1)	3979 (51.4)	4211 (54.6)	
Prediabetes	3049 (36.2)	2694 (36.2)	2673 (34.5)	2694 (34.9)	
Yes	2185 (25.9)	1398 (18.8)	1094 (14.1)	805 (10.4)	
Hypertension					<0.001
No	3811 (45.2)	4094 (55.0)	4832 (62.4)	5139 (66.7)	
Yes	4616 (54.8)	3353 (45.0)	2914 (37.6)	2571 (33.3)	
CVD					<0.001
No	6974 (82.8)	6671 (89.6)	7130 (92.0)	7263 (94.2)	
Yes	1453 (17.2)	776 (10.4)	616 (8.0)	447 (5.8)	

Abbreviations: n: sample size; BMI, body mass index (calculated as weight in kilograms divided by height in square meters); MET, metabolic equivalent; CVD, cardiovascular disease (suffering congestive heart failure, coronary heart disease, angina pectoris, heart attack, or stroke), Q, quartile.

## Data Availability

The datasets used and analyzed during the current study are available from the corresponding author on reasonable use.

## Supplementary Materials

The following supporting information can be downloaded at: https://www.mdpi.com/article/10.3390/ijerph20021398/s1, Figure S1: The association between physical activity levels and risk of diabetes after age stratification; Figure S2: The association between physical activity levels and risk of diabetes after sex stratification.

## Abbreviations

BMI, Body mass index; CI, confidence interval; CVD, cardiovascular disease; MET, metabolic equivalent; NHANES, National Health and Nutrition Examination Survey; OR, odds ratio; PAQ, Physical Activity Questionnaire.
