# Supplementary material for: Physical Activity Types, Physical Activity Levels and Risk of Diabetes in General Adults: The NHANES 2007–2018
Source: Int J Environ Res Public Health. 2023 Jan 12;20(2):1398. doi: 10.3390/ijerph20021398 (PMC9858810; doi:10.3390/ijerph20021398)
Supplement: Supplementary file 1 [file ijerph-20-01398-s001.zip › ijerph-2038687-supplementary.pdf]

Supplementary Figure S1. The association between physical activity levels and risk of diabetes after age stratification.

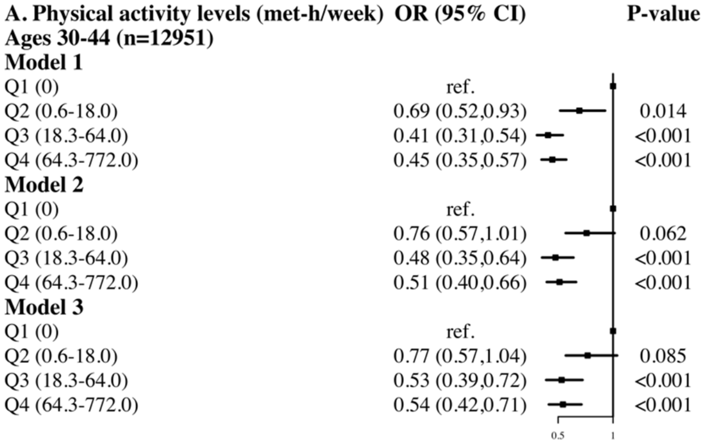

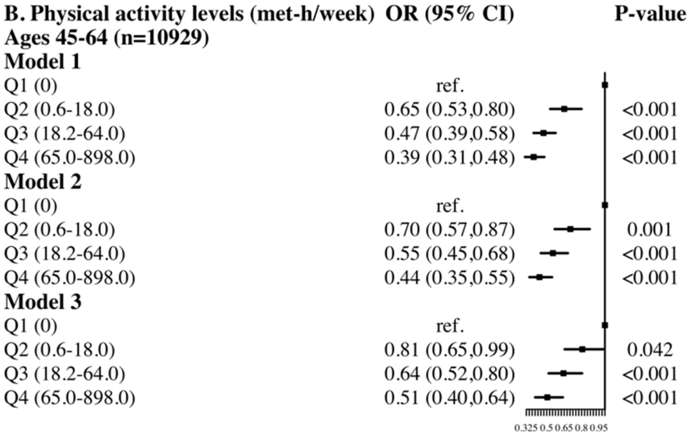

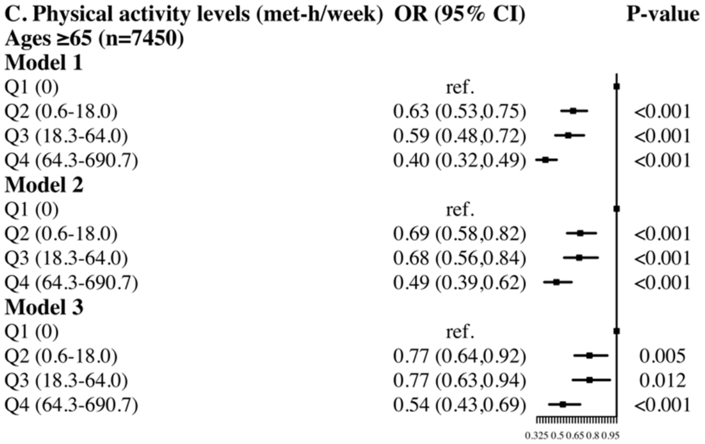

Model 1 adjusted for gender, age; Model 2 adjusted for gender, age, race, marital status, education level, occupation, smoke status, sedentary time. Model 3 gender, age, race, marital status, education level, occupation, smoke status, sedentary time, BMI, hypertension, CVD.

Supplementary Figure S2. The association between physical activity levels and risk of diabetes after sex stratification.

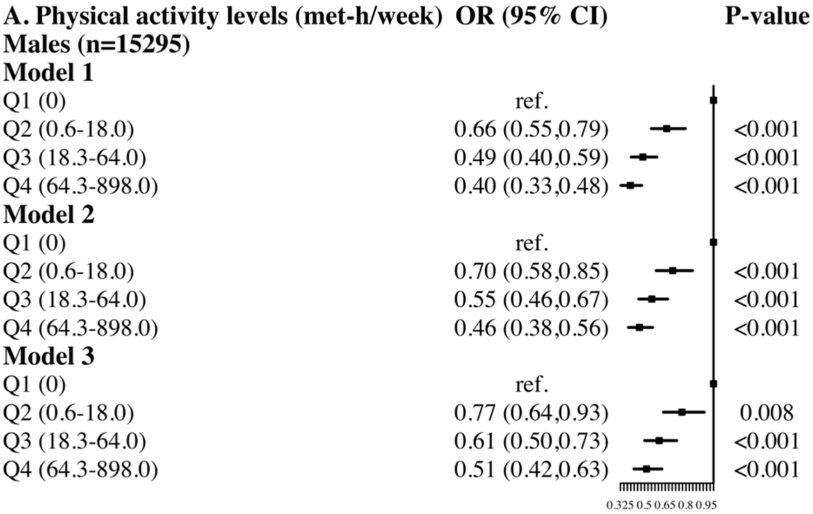

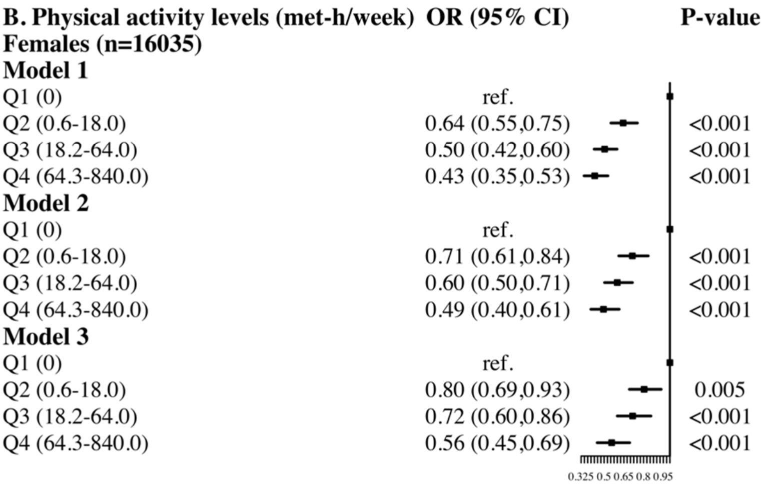

Model 1 adjusted for gender, age; Model 2 adjusted for gender, age, race, marital status, education level, occupation, smoke status, sedentary time. Model 3 gender, age, race, marital status, education level, occupation, smoke status, sedentary time, BMI, hypertension, CVD.
